# Nanozyme conjugated chitosan nanoparticles improve buffalo bull sperm cryopreservation by enhancing antioxidant defense and mitochondrial function

**DOI:** 10.3389/fvets.2025.1718467

**Published:** 2025-11-26

**Authors:** Ramya Ahmad Sindi, Mohammed A. Alfattah, Mahmoud A. E. Hassan, Ehab El-Haroun, Ahmed E. Noredlin, Sameh A. Abdelnour, Mahmoud Moussa

**Affiliations:** 1 Department of Clinical Laboratory Sciences, Faculty of Applied Medical Sciences, Umm Al-Qura University, Makkah, Saudi Arabia; 2 Department of Biology, College of Science, Jazan University, Jazan, Saudi Arabia; 3 Animal Production Research Institute, Agriculture Research Centre, Ministry of Agriculture, Dokki, Giza, Egypt; 4 Department of Integrative Agriculture, College of Agriculture and Veterinary Medicine, United Arab Emirates University, Al Ain, United Arab Emirates; 5 Department of Histology and Cytology, Faculty of Veterinary Medicine, Damanhour University, Damanhour, Egypt; 6 Department of Animal Production, Faculty of Agriculture, Zagazig University, Zagazig, Egypt; 7 Emirates Smart Camel Center, Umm Al Quwain, United Arab Emirates; 8 Department of Theriogenology, Faculty of Veterinary Medicine, Suez Canal University, Ismailia, Egypt

**Keywords:** buffalo, selenium conjugated chitosan nanoparticles, cryopreservation, sperm quality, oxidative stress

## Abstract

**Introduction:**

Cryopreservation is commonly used to preserve fertility and support genetic improvement in livestock. However, it often compromises sperm function and quality due to the excessive generation of oxidative stress. Nanozymes, a cutting-edge development in nanotechnology, offer a versatile and promising tool for mitigating oxidative stress caused by cryo-injury. This study targeted to assess the protective effects of selenium conjugated chitosan nanoparticles (SeCN; as a nanozyme) when added to semen freezing extenders in buffalo bulls.

**Methods:**

Semen samples were extended with 0 (SeCN0), 0.5 (SeCN0.5), 1 (SeCN1), or 2 (SeCN2) μg/mL SeCN, frozen at −196 °C, and assessed post-thaw for sperm quality, antioxidant status, mitochondrial activity and ultrastructural changes.

**Results:**

The SeCN supplementation significantly improved all post-thawed sperm parameters in a dose-dependent manner (*p* < 0.001). A significant linear increase (*p* < 0.01) was monitored in viability, sperm progressive motility, and plasma membrane integrity with increasing concentrations of SeCN supplementation. The SeCN2 group showed the highest percentages of sperm progressive motility, plasma membrane integrity, and viability related to SeCN groups (*p* < 0.05). Sperm abnormalities decreased linearly with SeCN supplementation, reaching the lowest rate in the SeCN2 group (*p* < 0.01 compared to other groups). Chromatin damage decreased significantly in a cubic manner in the SeCN1 and SeCN2 groups compared to the other groups (*p* < 0.01). For antioxidant status, the analysis showed a clear linear increase in both superoxide dismutase (SOD) levels and total antioxidant capacity (TAC) levels (*p* < 0.01). The SeCN2 group exhibited the highest SOD activity, while the SeCN1 and SeCN2 groups showed the highest TAC values (*p* < 0.01) compared to other groups. Additionally, glutathione peroxidase (GPx) activity in all SeCN-supplemented groups demonstrated a cubic increase (*p* < 0.01) compared to the control group. The SeCN2 group showed the most effective reduction in MDA levels, followed by the SeCN1 and SeCN0.5 groups (*p* < 0.001). Nitric oxide was significantly decreased in a linear manner by the addition of SeCN (*p* < 0.001). A significant cubic increase in mitochondrial membrane potential (MMP) was observed, with the addition of 1 or 2 μg of SeCN/mL (*p* < 0.001). Ultrastructural analysis via transmission electron microscopy confirmed improved preservation of acrosomal, mitochondrial, and plasma membrane integrity of buffalo spermatozoa.

**Discussion:**

At a concentration of 1 or 2 μg/mL, SeCN demonstrates potent cryoprotective effects by enhancing sperm function, reducing oxidative stress, and preserving mitochondrial activity and ultrastructure changes of sperm. Incorporating SeCN into semen extenders may improve cryosurvival in buffalo and represents a promising strategy for optimizing artificial insemination outcomes in livestock breeding programs.

## 1 Introduction

Water buffaloes (*Bubalus bubalis*) play a vital part in the global livestock economy, especially in Asia and Africa, where they are essential for milk and meat production and serve as draft animals in agriculture (1). Despite their significant economic and agricultural importance, buffalo herds encounter persistent and severe reproductive challenges that hinder genetic improvement and productivity (2). These inefficiencies arise from a combination of inherent biological limitations, such as a prolonged inter-calving period, silent estrus expression, and high embryonic mortality, exacerbated by suboptimal management practices (3).

Assisted reproductive technologies (ARTs), particularly semen cryopreservation, have emerged as crucial tools to overcome these challenges by facilitating the distribution of genetically superior males and preserving valuable genetic material (4, 5). Cryopreservation of sperm allows for long-term storage, genetic exchange across geographical boundaries, and improved reproductive efficiency in artificial insemination programs (6, 7). However, while this technique offers several advantages, it also introduces a series of physiological stresses that compromise sperm viability, functionality, and fertilizing potential (8, 9).

The primary concern associated with semen cryopreservation is oxidative stress, which arises from extreme synthesis of reactive oxygen species (ROS) throughout the thawing and freezing procedures (10). These ROS, when not adequately neutralized by the sperm’s intrinsic antioxidant defenses such as glutathione peroxidase (GPx), superoxide dismutase (SOD), and catalase (CAT), can initiate lipid peroxidation, damage mitochondrial membranes, impair DNA integrity, and ultimately reduce fertilization capacity (11–13). Oxidative damage also contributes to sperm morphological abnormalities, impaired motility, and decreased membrane integrity (13), particularly in species like buffalo, where spermatozoa are already highly susceptible to cold shock and osmotic stress (4, 9). To counteract these deleterious consequences, investigators have widely explored the use of exogenous antioxidants in cryopreservation media, specifically targeting enzymes like SOD and GPx.

Recently, nanotechnology-based approaches have obtained attention due to their capability to enhance bioavailability and targeted delivery of protective agents. Nanozymes are nanomaterials that mimic enzymatic functions have shown promising applications in biomedicine and reproductive biotechnology for their catalytic antioxidant properties and cellular permeability (14, 15). Among these, selenium nanoparticles (SeNPs) are particularly notable for their ability to emulate the function of GPx, a critical enzyme in ROS detoxification. The SeNPs have demonstrated strong antioxidative and anti-apoptotic properties and have been successfully applied to reduce cryo-damage in mammalian sperm (16, 17). Natural enzymes are vital protein catalysts in living systems, and their absence or deficiency can significantly disrupt biological functions (17), with the impact varying based on the specific enzyme and the degree of the shortfall. Nanozymes, nanoparticles engineered to mimic these natural enzymes, offer a promising alternative (18, 19). They are appealing for biological applications due to their ease of synthesis, enhanced stability, cost-effectiveness, and potent catalytic activity (19). The SeNPs are increasingly recognized as nanozymes, exhibiting various enzyme-like activities. Selenium is an essential trace element crucial for the function of many natural enzymes, particularly GPx and SOD, which plays a vital role in antioxidant defense (20). The SeNPs can mimic the activity of these selenium-containing enzymes. Some SeNPs can also convert superoxide radicals (O_2_−) into less harmful H_2_O_2_. In addition to these advancements, chitosan, a naturally derived, biodegradable polysaccharide, has emerged as a promising biopolymer for biomedical applications (21, 22). Its cationic nature enables it to interact with negatively charged sperm membranes, potentially stabilizing them during osmotic and thermal stress. Chitosan also possesses antioxidant and antimicrobial properties, which have been leveraged in various animal species to improve sperm quality during storage (23, 24). For instance, chitosan supplementation has been associated with enhanced motility and membrane integrity in rabbit (23), boer (25), ram (26) and bull (22) semen. However, conflicting findings exist regarding its compatibility with human sperm (27), necessitating further research, particularly in species with more delicate sperm like buffalo.

The integration of selenium with chitosan nanoparticles (SeCN) may offer synergistic benefits by combining the enzymatic antioxidant activity of selenium with the structural and bioactive properties of chitosan. This the combination has already demonstrated efficacy in other biomedical contexts, including oxidative stress mitigation and microbial inhibition (21, 28). Se-polydopamine nanozymes and chitosan-coated nanoparticles have demonstrated significant potential in restoring mitochondrial function and cellular antioxidant balance under stressful conditions (29).

Given the vulnerability of buffalo sperm to cryo-induced oxidative and structural damage, we hypothesized that SeCN supplementation could provide enhanced cryoprotection by preserving mitochondrial integrity, reducing oxidative stress, and maintaining chromatin stability. Therefore, the objective of the present study was to synthesize selenium conjugated chitosan nanoparticles (SeCN) and assess their dose-dependent effects on post-thaw sperm quality, antioxidant status, mitochondrial membrane potential, chromatin integrity, and ultrastructure in cryopreserved buffalo semen.

## 2 Materials and methods

### 2.1 Ethical statement

The study was reviewed and approved by the ZU-IACUC (Approval No. IACUC/2/F/25/2023). All methods complied with the ARRIVE guidelines, UK. Animals (Scientific Procedures) Act, 1986, EU Directive 2010/63/EU, and the National Research Council’s Guide for the Care and Use of Laboratory Animals (NIH Publications No. 8023, revised 1978).

### 2.2 Synthesis of selenium-chitosan nanoparticles

Sodium selenite (Na_2_SeO_3_) and Chitosan (Merck, Germany) were obtained and used as received. Commercial-grade chemicals sourced from Sigma-Aldrich were employed without further purification. These included chitosan with a low molecular weight range of 60–190 kDa and an 80–85% degree of deacetylation, and selenium tetrachloride (SeCl_4_) with a purity of ≥ 98%. For preparing selenium-chitosan nanoparticles (SeCN), we followed the methods of El-Megharbel et al. (21) and Song et al. (30).

Chitosan was dissolved in 1% acetic acid solution, filtered to eliminate impurities, and deprotonated with 2 M sodium hydroxide solution under stirring for 2 h. The resulting solution was then filtered, washed with distilled water, and adjusted to pH 7–8. After freezing for 72 h, the purified chitosan was used for the synthesis of SeCN (31). To prepare (SeCN), 3.0 mmol of purified chitosan was dispersed in 140 mL of distilled water. A solution of SeCl_4_ (Se (IV), 0.804 mmol) in 60 mL of distilled water was added dropwise to the chitosan solution under stirring for 40 min to ensure homogeneity. Subsequently, a thiourea solution (0.0901 mmol) in 100 mL of distilled water was added dropwise, followed by a 3-h cooling period. Finally, the SeCN sample was washed with ethanol (C_2_H_5_OH) and dried in an oven at 75 °C for 48 h. The morphology of synthesized selenium conjugated chitosan nanoparticles (SeCN) was characterized using Transmission Electron Microscopy (TEM) at 200 kV on a JEOL-JEM-2100 instrument (JEOL JEM-2100, Tokyo, Japan). Furthermore, the mean size, zeta potential, and polydispersity index (PDI) of SeCN were defined using a Zetasizer Nano ZS90 (Malvern Instruments, Malvern, UK).

### 2.3 Semen collection, experimental design

Five healthy Egyptian buffalo bulls were picked for this research and acquired from the International Livestock Management Training Center (ILMTC) in Mahalt Moussa, Sakha, Kafer El Sheikh. They had an average age of 5–6 years and weighed between 520 and 580 kg. Semen samples (*n* = 40) were collected from the bulls utilizing the artificial vagina technique. Forty ejaculates were obtained over seven consecutive weeks from five bulls. The bulls were housed individually in separate pens and fed a consistent diet of farm fodder, including apple pomace, vitamin premix, maize silage, basic minerals, and haylage according to the NRC recommendations (32). They had unrestricted access to fresh water. Only ejaculates meeting the following criteria were selected, pooled, and used for freezing assessment: progressive motility exceeding 75%, viability greater than 80%, Sperm concentration of at least 500 × 10^6^/mL, and abnormality rate less than 15%. A freezing extender was prepared based on the protocol of (4) with minor modifications, adhering to standard procedures at the ILMTC. The extender comprised 7.0 mL glycerol, 1.675 g citric acid, 1.25 g fructose, 3.028 g Tris, 20 mL egg yolk, 100 μg/mL streptomycin, and 100 IU/mL penicillin in double-distilled water, adjusted to a final volume of 100 mL. Fresh semen was divided into four equal fractions and subjected to different treatments prior to cryopreservation. The semen samples were supplemented with 0 (SeCN0), 0.5 (SeCN0.5), 1 (SeCN1) and 2 (SeCN2) μg/mL of selenium conjugated chitosan nanoparticles. Each fraction was packaged into 0.25 mL straws (30×10^6^), equilibrated for 2 h at 4 °C, and then subjected to vapor phase freezing for 10 min above liquid nitrogen (−196 °C) before immersion in liquid nitrogen. The semen quality and oxidative stress of post-thawed semen were assessed following cryopreservation.

### 2.4 Evaluating semen quality

#### 2.4.1 Progressive motility

To determine sperm motility, a droplet (5–10 μL) of post-thawed semen was placed on a 37 °C slide, covered with a coverslip, and examined using a phase-contrast microscope (100×, Leica DM 500, Schweiz). The proportion of progressively motile sperm was then determined by counting sperm in a minimum of five different fields of view, maintained at 37 °C, with a reported validity of ±5%. Subjective motility assessments were recorded.

#### 2.4.2 Sperm viability and abnormality

Sperm viability was assessed using the eosin-nigrosine staining method (33). A small semen sample was mixed with eosin-nigrosine solution on a slide, allowed to stand for 2–3 min, and then air-dried. Under a light microscope at 400x magnification (Leica DM 500, Switzerland), viable sperm remained unstained, while non-viable sperm-stained red. Sperm cells with morphologic irregularities (head and tail defects) were also documented.

#### 2.4.3 Plasma membrane integrity

The integrity of the plasma membrane was assessed using the hypo-osmotic swelling test (HOST) (34). Thawed semen (10 μL) was incubated in a hypo-osmotic solution containing fructose and sodium citrate at 37 °C. Sperm that showed swelling were classified as having intact plasma membranes. The observations were conducted using a phase-contrast microscope (Leica DM 500, Switzerland) at 400x magnification.

#### 2.4.4 Sperm chromatin damage

Chromatin damage was judged using the toluidine blue staining method (35) with modifications. Sperm cells were fixed, hydrolyzed, and stained with toluidine blue in McIlvaine buffer (pH 4.0). Under a light microscope at 1000x magnification, sperm with normal chromatin stained green to light blue, while those with damaged chromatin-stained dark blue to violet. A total of 300 sperm cells were analyzed per group.

### 2.5 Mitochondrial membrane potential

The lipophilic cation JC-10 was utilized to evaluate the mitochondrial activity of spermatozoa using the JC-10 (Flow Cytometry, ab112133) Mitochondrial Membrane Potential Assay Kit (36). Frozen–thawed semen samples were diluted to 1 × 10^6^ sperm/mL. After washing with PBS and centrifugation, the pellet was resuspended in 500 μL of JC-10 and incubated for 1 h at 37 °C. Subsequently, the cells were centrifuged, resuspended in PBS, and analyzed by flow cytometry. The percentage of sperm exhibiting orange fluorescence (JC-10 aggregates), revealing of high mitochondrial membrane potential (MMP), was quantified using emission filters of 535 nm.

### 2.6 Assessing oxidants-antioxidant status

Post-thawed semen was centrifuged at 1600 x g for 5 min, and the supernatant was collected. Cellular protein was extracted using 1% Triton X-100, followed by centrifugation at 4000 x g for 30 min. Superoxide dismutase (SOD; A001-1-2) activity (U/mL), glutathione peroxidase (GPx; A005-1-2), and total antioxidant capacity (TAC, mmol/L, A015-1-1) were measured spectrophotometrically using ELISA kits (Jiancheng Bioengineering Institute) according to the method described in reference (37). Malondialdehyde (MDA, A003-4-1) levels (nmol/mL), an indicator of lipid peroxidation, were determined using the thiobarbituric acid (TBA) assay with kits from Jiancheng Bioengineering Institute, following the manufacturer’s instructions and the method outlined in reference (4). Nitric oxide was assessed using the method described in reference (38).

### 2.7 Sperm ultrastructure

Sperm ultrastructural changes were assessed using TEM following the method described by Khalil et al. (8). Post-thawed semen (500 μL) was fixed in 4% glutaraldehyde in DPBS at 4 °C for 2 h, then washed and post-fixed in 1% osmium tetroxide at room temperature for 1 h. The subsequent steps involved dehydration in graded ethanol, embedding in propylene oxide and Epon resin, and ultrathin sectioning (60–70 nm). The sections were examined using a JEOL 2100 TEM at 80 kV to identify any treatment-related alterations in sperm head (acrosome, chromatin, nucleus, plasma membrane) and mid-piece mitochondrial morphology.

### 2.8 Data statistical analysis

The Shapiro–Wilk and Levene tests were used to confirm the homogeneity and normality of variance. The MIXED model procedure of the Statistical Analysis System (SAS, PROC MIXED; Institute, 2012) was employed to assess different sperm quality and oxidant-antioxidant status. Duncan’s multiple range test was conducted as a post-hoc analysis to investigate individual group differences beyond the overall ANOVA result. To determine a possible dose–response curve, the response of each dependent variable to various levels of SeCN (0, 0.5, 1, and 2 mL SeCN/mL extender) was evaluated using orthogonal contrast statements for linear, cubic and quadratic responses. Dose–response curves were fitted using the suitable regression equation to determine the optimal SeCN supplementation dose using GraphPad prism (version 8). A *p*-value of <0.05 is judged statistically significant.

## 3 Results

### 3.1 Characterization of selenium-chitosan nanoparticles

**Figure 1:**
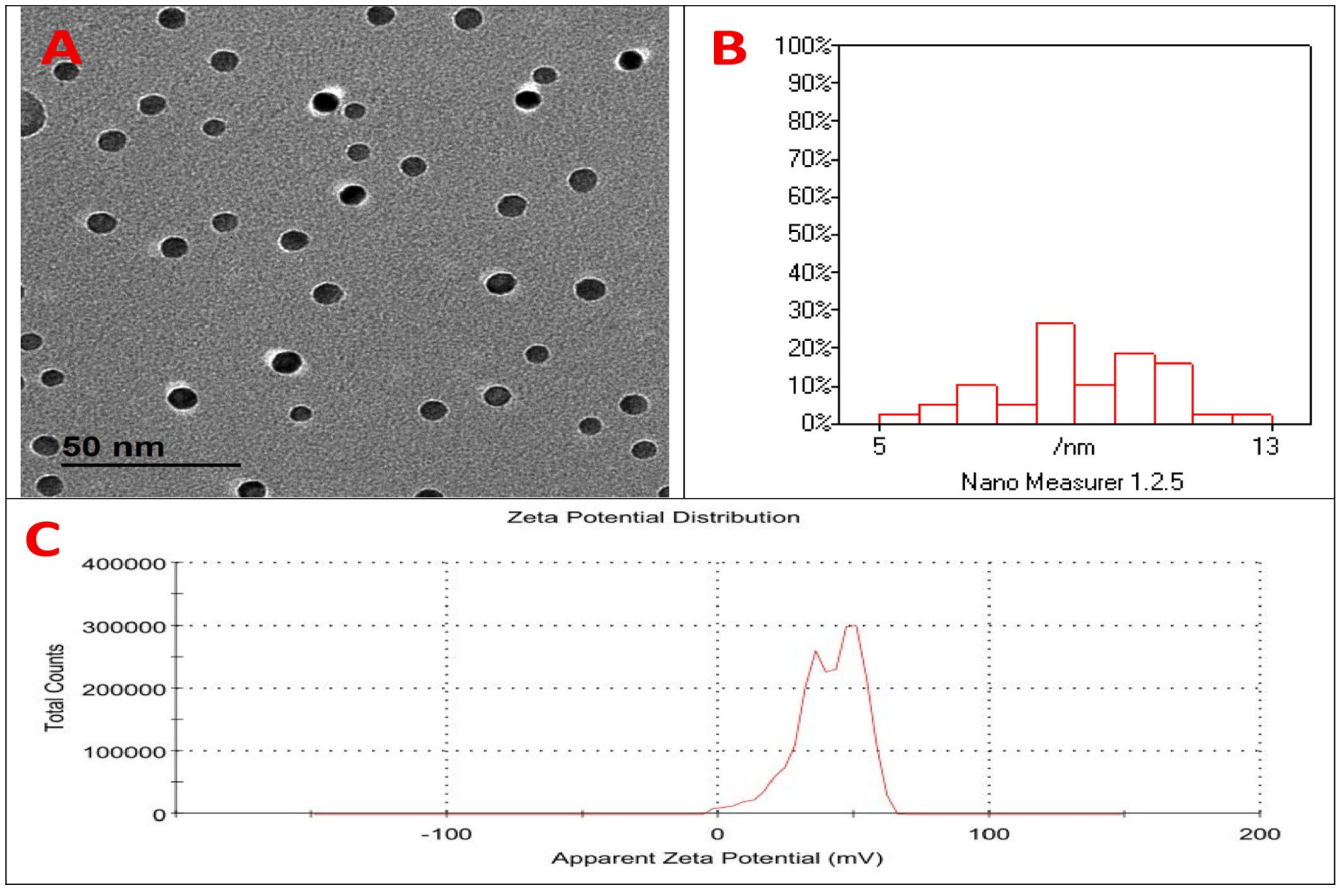
Characterization of selenium-chitosan nanoparticles (SeCN). **(A)** Transmission electron microscopy (TEM) image showing spherical, well-dispersed SeCN with uniform morphology. **(B)** Dynamic light scattering (DLS) analysis indicating average particle size of 5–13 nm. **(C)** Zeta potential measurement showing a mean surface charge of +40.3 mV, indicating good colloidal stability.

The characterization of selenium chitosan nanoparticles (SeCN) is illustrated in Figure 1. Figure 1A displays spherical, well-dispersed SeCN with a uniform morphology observed through Transmission Electron Microscopy (TEM). The particle size distribution histogram indicates a dominant range of 5–13 nm (Figure 1B) using Dynamic Light Scattering (DLS) analysis, while the Zeta potential distribution peaks at 40 mV (Figure 1C) indicating good colloidal stability.

### 3.2 Impacts on the post-thawed sperm attributes

**Figure 2:**
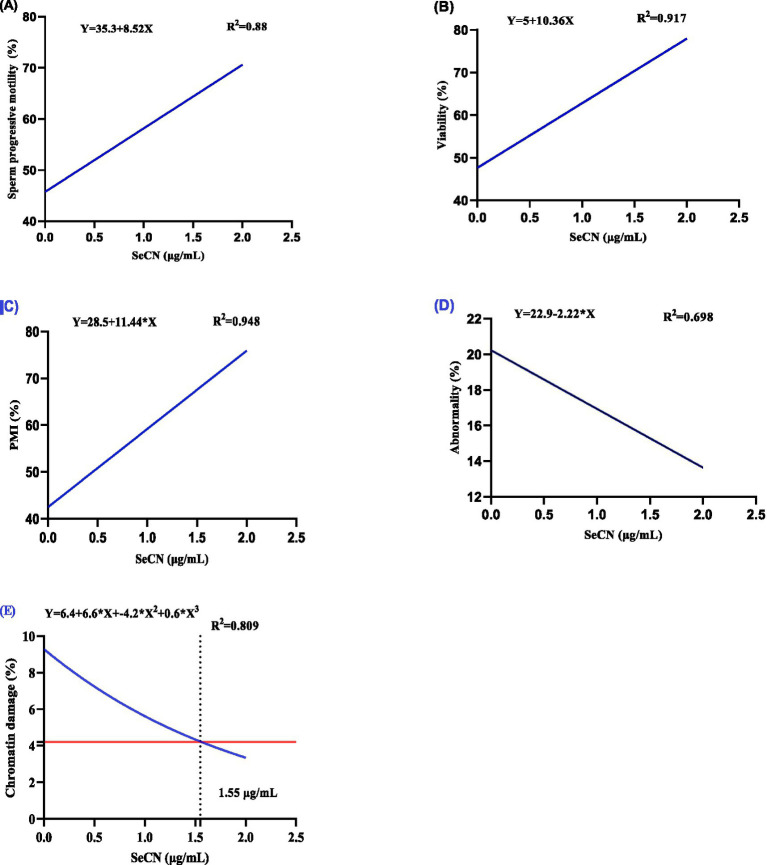
A multinomial regression assessment between selenium-chitosan nanoparticles (SeCN, 0, 0.5, 1 and 2 μg/mL) and various post-thawing sperm attributes including **(A)** Sperm progressive motility (%), **(B)** sperm viability (%), **(C)** Plasma membrane integrity (%), **(D)** sperm abnormality (%), and **(E)** Chromatin damage. The *p*-values of the ANOVA statistical analysis and the linear (Lin), quadratic (QUA) and cubic (CUB) effects of SeCN adding levels were tested via polynomial contrasts.

Table 1 demonstrates the dose-dependent effects of SeCN on post-thawed sperm quality parameters. A significant linear increase (*p* < 0.01) was observed in sperm motility (SPM, *R*^2^ = 0.88, Figure 2A), viability (*R*^2^ = 0.917, Figure 2B), and PMI (*R*^2^ = 0.948, Figure 2C) with increasing concentrations of SeCN supplementation.

**Table 1:** The effects of different doses of selenium-chitosan nanoparticles (SeCN) at 0, 0.5, 1, and 2 μg/mL added to the freezing extender on the post-thawed sperm characteristics of buffalo.

Treatment	Sperm characteristics (%)				
	SPM	Viability	PMI	Abnormality	CHROM
SeCN0	43.00^d^	45.80^d^	40.40^d^	21.20^a^	9.20^a^
SeCN0.5	53.00^c^	53.80^c^	49.20^c^	17.40^b^	7.80^a^
SeCN1	62.00^b^	68.60^b^	65.80^b^	16.80^b^	3.80^b^
SeCN2	71.00^a^	78.80^a^	75.60^a^	14.60^c^	3.60^b^
SEM	2.53	3.05	3.210	0.650	0.240

*p* values					
ANOVA	<0.001	<0.001	<0.001	<0.001	<0.001
LIN	<0.001	<0.001	<0.001	<0.001	<0.001
QUA	0.785	0.541	0.710	0.307	0.286
CUB	0.903	0.167	0.025	0.176	0.018

Sperm progressive motility (SPM), plasma membrane integrity (PMI), sperm chromatin damage (CHROM), linear (Lin), quadratic (QUA) and cubic (CUB) effects. SeCN0, SeCN0.5, SeCN1, SeCN2 indicate freezing extender supplemented with 0, 0.5, 1 or 2 μg of selenium chitosan nanoparticles (SeCN) /mL. Results are presented as means± pooled standard error (SEM). ^a,b,c,d^ Means within a column without a common superscript letter differ at *p* < 0.05.

The SeCN2 group exhibited the highest percentages of SPM, PMI, and viability compared to all other groups (*p* < 0.05). Conversely, sperm abnormalities (*R*^2^ = 0.698, Figure 2D) decreased linearly with SeCN supplementation (*p* < 0.01 compared to other groups), reaching the lowest rate in the SeCN2 group. The results of sperm abnormalities did not statistically differ between the SeCN0.5 and SeCN1.0 groups (*p* > 0.05). Chromatin damage decreased significantly in a cubic manner (*R*^2^ = 0.809) in the SeCN1 and SeCN2 groups compared to other groups, with an estimated optimal dose of 1.7 μg/mL (Figure 2E).

### 3.3 Impacts on antioxidant and oxidative stress in post-thawed semen

**Figure 3:**
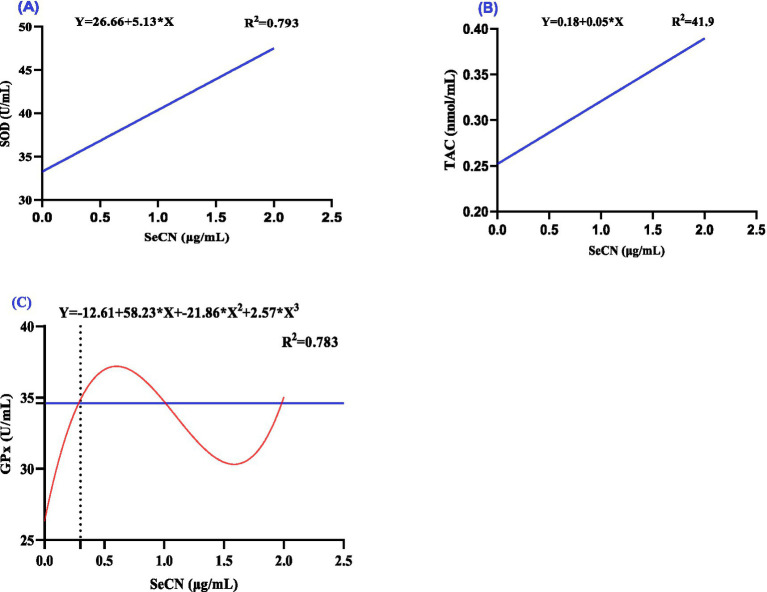
A multinomial regression assessment between selenium-chitosan nanoparticles (SeCN, 0, 0.5, 1 and 2 μg/mL) and antioxidant status of post-thawed sperm. **(A)** Superoxide dismutase (SOD, U/mL), **(B)** Total antioxidant capacity (TAC, nmol/mL), and **(C)** Glutathione peroxidase (GPx, U/mL). The *p*-values of the ANOVA statistical analysis and the linear (Lin), quadratic (QUA) and cubic (CUB) effects of SeCN adding levels were tested via polynomial contrasts.

**Figure 4:**
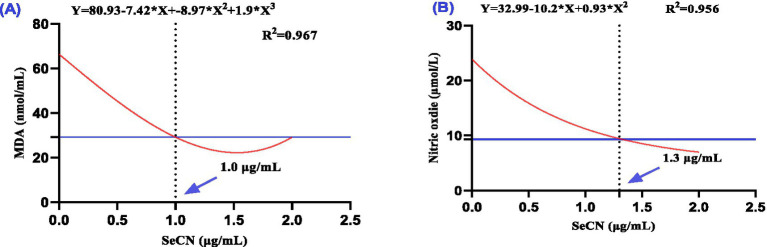
A multinomial regression assessment between selenium-chitosan nanoparticles (SeCN, 0, 0.5, 1 and 2 μg/mL) and oxidative stress markers in post-thawed buffalo such as **(A)** Malondialdehyde (MDA, nmol/mL) and **(B)** Nitric oxide (NO, μmol/L). The *p*-values of the ANOVA statistical analysis and the linear (Lin), quadratic (QUA) and cubic (CUB) effects of SeCN adding levels were tested via polynomial contrasts.

The effect of increasing SeCN concentrations in the freezing extender on antioxidants (Figure 3) and oxidative (Figure 4) markers in seminal plasma were analyzed using polynomial regression (Table 2). The analysis demonstrated a linear increase in both SOD levels (R^2^ = 0.793, Figure 3A) and TAC levels (*R*^2^ = 0.419, Figure 3B). The SeCN2 group had the highest SOD activity, while the SeCN1 and SeCN2 groups had the greatest TAC values (Table 2). Furthermore, GPx activity in all SeCN-supplemented groups displayed a cubic increase (*R*^2^ = 0.783, Figure 3C) compared to the control group.

**Table 2:** Impacts of various doses of selenium-chitosan nanoparticles (SeCN, 0, 0.5, 1 and 2 μg/mL) added to freezing extender on antioxidants and oxidative stress of post-thawed sperm attributes of buffalo spermatozoa.

Treatment	Antioxidant markers			Oxidative markers	
	SOD (U/mL)	TAC (nmol/mL)	GPx (U/mL)	NO (nmol/mL)	MDA (nmol/mL)
SeCN0	31.85^d^	0.21^c^	26.32^b^	23.88^a^	66.43^a^
SeCN0.5	39.03^c^	0.29^b^	36.67^a^	15.86^b^	45.38^b^
SeCN1	44.32^b^	0.36^a^	34.72^a^	11.26^c^	29.16^c^
SeCN2	47.97^a^	0.35^a^	35.03^a^	6.99^d^	29.16^c^
SEM	1.482	0.020	1.086	1.467	3.570

*p* values					
ANOVA	<0.001	0.021	<0.001	<0.001	<0.001
LIN	<0.001	0.004	0.001	<0.001	<0.001
QUA	0.132	0.227	0.014	0.010	<0.001
CUB	0.965	0.677	0.014	0.294	0.088

Superoxide dismutase (SOD), total antioxidant capacity (TAC), nitric oxide (NO), glutathione peroxidase (GPx), and malondialdehyde (MDA), linear (Lin), quadratic (QUA) and cubic (CUB) effects. SeCN0, SeCN0.5, SeCN1, SeCN2 indicate freezing extender supplemented with 0, 0.5, 1 or 2 μg of selenium chitosan nanoparticles (SeCN) /mL. Results are presented as means± pooled standard error (SEM). ^a,b,c,d^ Means within a column without a common superscript letter differ at *p* < 0.05.

The SeCN2 group showed the most effective reduction in MDA levels, followed by the SeCN1.0 and SeCN0.5 groups, which exhibited progressively less reduction (Table 2). Cubic regression analysis showed that an optimal SeCN concentration of 1 μg/mL was found to minimize lipid peroxidation (MDA, Figure 4A, *R*^2^ = 0.967). Similarly, an optimal SeCN concentration of 1.3 μg/mL was observed for reducing nitric oxide (NO) levels (Figure 4B, *R*^2^ = 0.956). In contrast, nitric oxide levels decreased linearly with increasing SeCN concentration in a dose-dependent manner compared to the control (*R*^2^ = 0.966, Figure 4B).

### 3.4 Impacts on mitochondria activity

**Figure 5:**
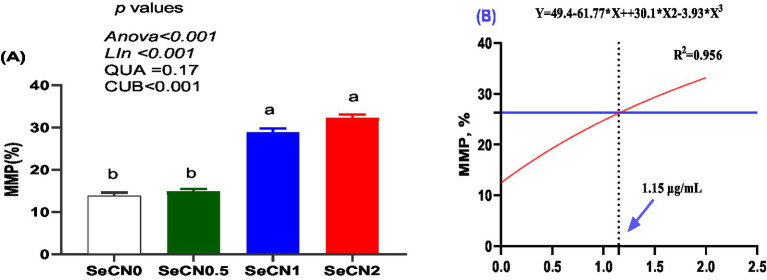
Mitochondrial membrane potential (MMP) of post-thawed buffalo sperm assessed by JC-1 staining and flow cytometry. **(A)** Percentage of sperm with high MMP (orange fluorescence). **(B)** A multinomial regression assessment between selenium-chitosan nanoparticles (SeCN, 0, 0.5, 1 and 2 μg/mL) and MMP. The *p*-values of the ANOVA statistical analysis and the linear (Lin), quadratic (QUA) and cubic (CUB) effects of SeCN adding levels were tested via polynomial contrasts.

A significant cubic increase in mitochondrial membrane potential (MMP) was observed, with the SeCN1 and SeCN2 groups displaying the highest values (Figure 5A). The optimal SeCN concentration for MMP (*R*^2^ = 0.956) was determined to be 1.15 μg/mL (Figure 5B), indicating the dose at which the greatest MMP was achieved.

### 3.5 Subcellular structural alterations of buffalo spermatozoa

**Figure 6:**
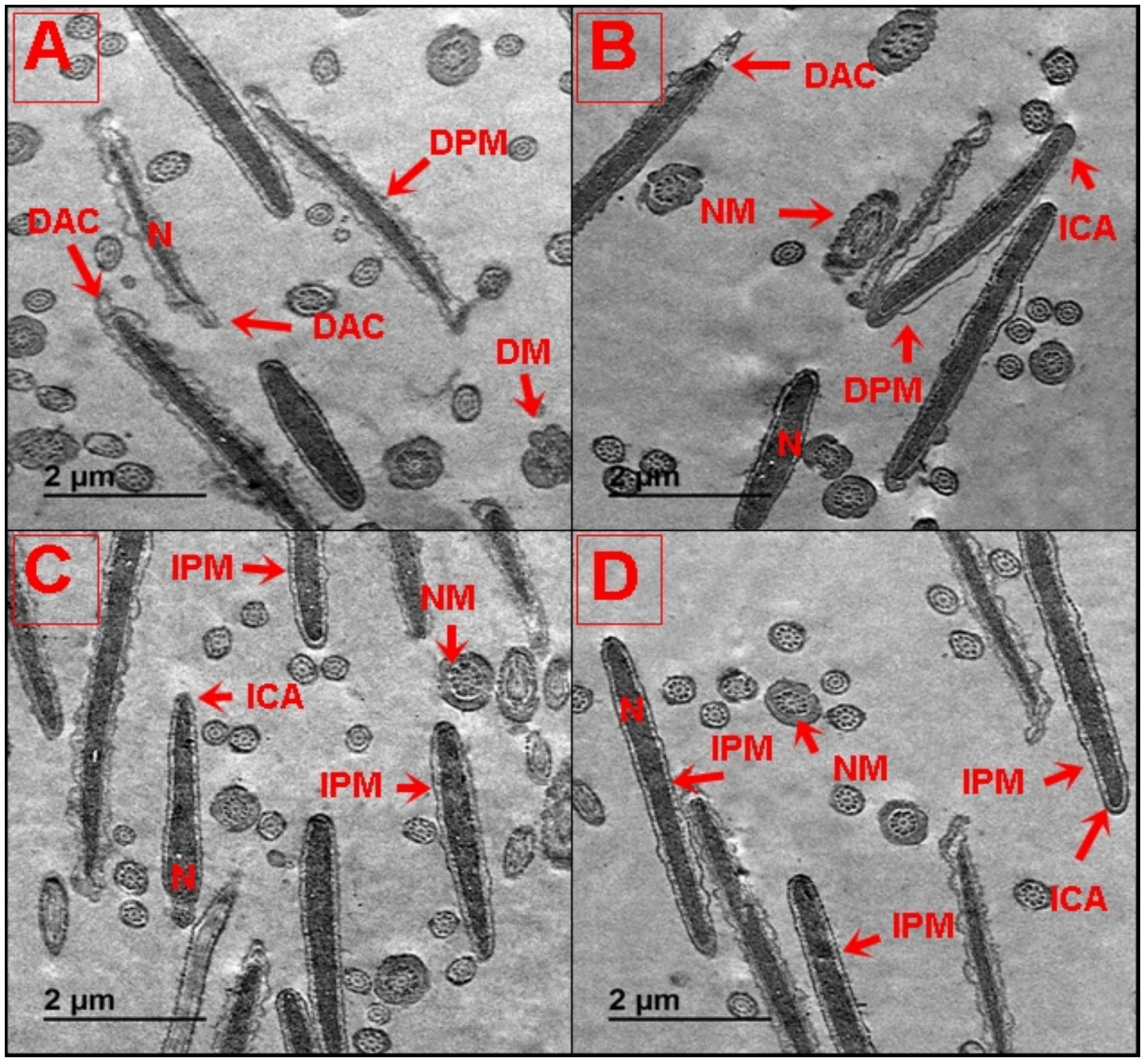
**(A–D)** Ultrastructural analysis of buffalo spermatozoa by TEM following treatment with SeCN. **(A)** Control group (0 μg/mL SeCN) showing damaged plasma membrane and swollen mitochondria, **(B)** 0.5 μg/mL SeCN showing intact plasma membrane, condensed acrosome, and preserved mitochondrial cristae, **(C)** 1 μg/mL SeCN exhibiting similar structural preservation, and **(D)** 2 μg/mL SeCN with occasional acrosomal detachment, suggesting potential toxicity at higher doses. Labels denote: N (nucleus), DPM (damaged plasma membrane), IPM (intact plasma membrane), NM (normal mitochondria), DM (damaged mitochondria), ICA (intact acrosome), and DAC (damaged acrosome). Scale bar = 500 nm.

To gain detailed insight into the subcellular structural alterations of buffalo spermatozoa post-cryopreservation, TEM analysis was performed after treatment with different concentrations of SeCN (Figures 6A–D). Buffalo spermatozoa in the control group (SeCN0) showed significant subcellular structural damage, including tail and neck abnormalities, a damaged plasma membrane (DPM), a damaged acrosome (DAC), damaged mitochondria (DM), and altered acrosomal membranes (Figure 6A). However, supplementing the cryomedia with SeCN at 0.5 μg/mL (Figure 6B) and 1 μg/mL (Figure 6C) demonstrated a protective effect on buffalo spermatozoa subcellular structures. This resulted in reduced nuclear damage, normal mitochondrial and nuclear morphology, and preserved acrosome and plasma membrane integrity. Exposure to 2 μg/mL SeCN (Figure 6D) resulted in protective effects on buffalo spermatozoa, reducing damage to the acrosome and plasma membrane. This was observed through a decrease in damaged mitochondria and a more heterogeneous population of intact acrosomes compared to damaged acrosomes.

## 4 Discussion

The use of selenium conjugated chitosan nanoparticles (SeCN) as a new cryoprotectant in buffalo semen cryopreservation represents a major breakthrough in reproductive biotechnology. This study presents strong evidence that supplementing with SeCN at optimal concentrations (1–2 μg/mL) enhances post-thaw sperm function, boosts antioxidant defense mechanisms, preserves mitochondrial activity, and maintains ultrastructural integrity. These findings not only validate the effectiveness of SeCN at a cellular level but also highlight its potential application in livestock artificial insemination programs. Cryopreservation-induced oxidative stress remains a major barrier to preserving sperm functionality, particularly in species like buffalo, which are highly susceptible to lipid peroxidation due to the high content of polyunsaturated fatty acids in their sperm membranes. The study demonstrated a significant reduction in lipid peroxidation markers such as MDA and NO, alongside elevated levels of key antioxidant enzymes (SOD, GPx, and TAC), indicating enhanced redox homeostasis. These findings are consistent with previous reports showing selenium nanoparticles that possess GPx-like activity that neutralizes harmful peroxides (14, 16).

Nanozymes, with their potent catalytic properties, hold great promise in restoring cellular homeostasis and enhancing defensive responses in challenging conditions. Their ability to mimic natural enzymes, along with their stability and cost-effectiveness, makes them highly advantageous (15, 27). The bioactivity of SeCN seems to arise from its dual mechanism: selenium’s potent antioxidant properties and chitosan’s membrane-stabilizing and antimicrobial attributes (39). Chitosan’s positive surface charge allows interaction with negatively charged sperm membranes, potentially forming a protective barrier that reduces osmotic and thermal stress during freeze–thaw cycles (23, 40). Additionally, transmission electron microscopy confirmed that SeCN-treated sperm maintained acrosomal integrity, plasma membrane continuity, and mitochondrial morphology essential factors for successful fertilization.

Sperm motility is a crucial parameter used to assess the male gamete’s fertilization competence (5). Viability assays are critical for fertility, serving to confirm the percentage of live sperm and differentiate between immotile and truly dead cells (8). The integrity of the sperm plasma membrane is crucial for quality, as its proper function is essential for motility, capacitation, and ultimately, fusion with the oocyte, directly influencing fertilization success (34). Analyzing chromatin damage provides the necessary profile of DNA integrity within the sperm (35). In this study, SeCN at 2 μg/mL exhibited the highest values of sperm motility, viability, and PMI, followed by the SeCN1 group (*p* < 0.001). Conversely, sperm abnormalities decreased linearly with SeCN supplementation, reaching the lowest rate in the SeCN2 group. These results are consistent with previous findings in reference (8), which showed that adding selenium nanoparticles significantly improved post-thawed sperm quality in bulls. In buffaloes, the addition of metabolic nanoparticles significantly improved progressive motility, plasma membrane integrity, and viability (9) by reducing oxidative stress. Additionally, adding selenium nanoparticles at a concentration of 2 μg/mL to significantly enhanced sperm motility, viability, PMI, DNA integrity, acrosome integrity, and MMP of boar semen (41).

The study showed that SeCN improved sperm quality by reducing oxidative stress and enhancing mitochondrial function. Specifically, SeCN1 and SeCN2 groups had significantly higher MMP, indicating better sperm bioenergetic health. This suggests that SeCN not only protects mitochondria from damage but also maintains ATP production, crucial for sperm motility and fertilization (36, 42). Overall, SeCN appears to stabilize sperm energy metabolism by reducing mitochondrial ROS and preserving membrane potential. Multiple studies have revealed that the addition of selenium nanoparticles can reduce the levels of MDA and NO and enhance the antioxidant status in post-thawed semen in buffalo (8) and in boar (41), as reported in this study. In a study, it was shown that adding chitosan nanoparticles significantly improved the antioxidant status and reduced the levels of MDA compared to the control group (26).

The protective effects of SeCN are largely attributed to the activation of the nuclear factor erythroid 2–related factor 2 (NRF2) signaling pathway, which is the master regulator of cellular antioxidant defenses. Selenium compounds stimulate NRF2 translocation to the nucleus to promote the transcription of key antioxidant enzymes (e.g., GPX4, SOD, HO-1) (14, 43). As GPX4 is critical for preventing lipid peroxidation in sperm membranes, this upregulation of endogenous antioxidant defenses explains the observed increases in redox biomarkers and mitochondrial membrane potential (17, 43). Moreover, SeCN’s antimicrobial potential offers added value, especially in the context of rising concerns over antibiotic resistance in semen extenders. Chitosan’s bactericidal properties and selenium’s potential to inhibit microbial growth may enable development of antibiotic-free semen preservation systems, aligning with global efforts to reduce antimicrobial residues in food animals (44, 45).

To gain more insight into how SeCN preserves the sperm structure, TEM was performed to clarify the ultrastructure changes of sperm. This study found that SeCN0 showed extensive damage in the tail, neck, plasma membrane (DPM), acrosome (DAC), and mitochondria (DM). However, supplementation with 0.5 μg/mL and 1 μg/mL SeCN demonstrated a protective effect, preserving the integrity of the nucleus, mitochondria, acrosome, and plasma membrane. The highest concentration of 2 μg/mL SeCN also showed protective effects, particularly reducing damage to the acrosome and plasma membrane and displaying a more heterogeneous population of intact acrosomes. These results are consistent with many previous studies that indicated the addition of selenium nanoparticles (8, 41) or chitosan nanoparticles (26) substantially enhanced the sperm ultrastructure. Moreover, chitosan/selenium inhibits oxidative stress in tissue damage induced by diclofenac sodium in rats (21). Selenium protects cells from oxidative stress, enhancing mitochondrial activity and maintaining DNA integrity in sperm. Nanozymes enhance sperm quality by reducing oxidative stress, protecting membrane integrity, and preserving mitochondrial function (20, 46). While the study shows promising results, its reliance on *in vitro* assays restricts the generalization of findings to actual reproductive outcomes. This study found significant improvements in sperm motility, viability, and chromatin integrity in all SeCN groups compared to control groups. Further clarification is needed, especially through *in vivo* experiments, to validate these results (47, 48). Therefore, future research should focus on controlled fertility trials to evaluate pregnancy rates, embryo development, and the health of offspring after SeCN-treated inseminations. SeCN showed a dose-dependent improvement in most parameters, but the highest dose tested (2 μg/mL) resulted in marginal ultrastructural compromise. This suggests a narrow therapeutic window and raises concerns about potential reductive stress from excessive antioxidant supplementation. Similar effects have been seen with other antioxidants, where high levels can impair rather than enhance sperm function (10). To provide a broader context for SeCN, it is essential to compare it with other nano-antioxidants being studied. Zinc oxide, cerium oxide, and carbon dots have shown promise in sperm cryopreservation, but they have limitations like toxicity at high doses or inconsistent stability. In contrast, SeCN offers a combination of effectiveness, biocompatibility, and antimicrobial properties, making it a promising candidate for further research (16, 24).

## 5 Conclusion

Our findings strongly suggest that nanozyme-chitosan nanoparticles (SeCN) significantly improve the cryopreservation of buffalo sperm. This is achieved through various mechanisms, including antioxidant protection, stabilization of mitochondria, and preservation of cellular membranes. The addition of 1 or 2 μg/mL SeCN to buffalo freezing media resulted in increased progressive motility and viability, maintained plasma membrane integrity, and reduced chromatin damage and abnormalities. Furthermore, SeCN improved mitochondrial membrane potential and key antioxidant markers (SOD, GPx, and TAC), while decreasing NO and MDA levels. However, for SeCN to become a practical tool for livestock breeding, several research gaps need to be addressed. These include validating *in vitro* findings through *in vivo* fertility trials, conducting comprehensive economic analyses for field implementation, performing rigorous long-term biosafety assessments, and engaging with regulatory bodies for nanoparticle approval. Overcoming these challenges could establish SeCN as an innovative solution for artificial insemination protocols, applicable to a wide range of livestock species and breeding systems. Further studies are needed to explore the specific mechanisms through which nanozymes enhance antioxidant defense, utilizing transcriptomic and proteomic tools.

## Data Availability

The raw data supporting the conclusions of this article will be made available by the authors, without undue reservation.
